# Protective Effects of Vitamin C against Neomycin-Induced Apoptosis in HEI-OC1 Auditory Cell

**DOI:** 10.1155/2022/1298692

**Published:** 2022-05-11

**Authors:** Liang Gong, Biao Chen, Jingyuan Chen, Yongxin Li

**Affiliations:** Department of Otorhinolaryngology Head and Neck Surgery, Beijing Tongren Hospital, Capital Medical University, Beijing 100005, China

## Abstract

Ototoxic hearing loss results from hair cell death via reactive oxygen species (ROS) overproduction and consequent apoptosis. We investigated the effects of vitamin C (VC) on neomycin-induced HEI-OC1 cell damage, as well as the mechanism of inhibition. HEI-OC1 cells were treated with neomycin or with vitamin C (VC). The results indicated that VC had a protective effect on neomycin-induced HEI-OC1 cell death. Mechanistically, VC decreased neomycin-induced ROS generation, suppressed cell death, and increased cell viability. VC inhibited neomycin-induced apoptosis, ameliorated neomycin reduced antiapoptotic Bcl-2 expression, and suppressed neomycin increased expression of proapoptotic Bax, caspase-3 cleavage and caspase-8. TUNEL labeling demonstrated that VC blocked neomycin-induced apoptosis. Further study revealed that the effect of VC on neomycin-induced hair cell death was through interference with JNK activation and p38 phosphorylation. These results indicate that VC via suppressed ROS generation, which inhibited cell death by counteracting apoptotic signaling induced by neomycin in cells. Hence, VC is a potential candidate for protection agent against neomycin-induced HEI-OC1 cell ototoxicity.

## 1. Introduction

Hearing loss has profound impacts on individuals by, for example, causing communication problems, social isolation, and reduced quality of life. There are many causes of hearing loss, including ototoxic drug-induced, noise-induced, and age-related hearing loss. According to the Centers for Disease Control and Prevention (CDC) report in 2012, approximately 15% of American adults have hearing loss [1]. Sensorineural hearing loss in mammals is incurable because hair cells cannot be regenerated once they are lost.

Neomycin is a type of aminoglycoside used in the treatment of hepatic encephalopathy and surgical prophylaxis [2] in the clinic. However, the adverse effects of aminoglycoside antibiotics can lead to ototoxicity with significant and permanent hearing loss. ROS act as agents of aminoglycoside induced ototoxicity and are the main initiators of aminoglycoside-induced hearing loss [3]. ROS triggers various cell death mechanisms that include caspase-dependent or independent apoptosis and necrosis [4, 5]. In addition to apoptosis induced by ROS, neomycin reportedly induces hair cell apoptosis via p38, MAPK, and JNK activation, as well [6–8].

Currently, there are no therapeutic methods to restore lost hair cells; therefore, searching for a new method to reduce the ototoxic effect on hair cells to prevent hearing loss is essential. In recent years, a diverse assortment of agents with protective action against neomycin-induced ototoxicity have been reported. Several studies have investigated and indicated that certain antioxidants may protect against neomycin-induced hair cell death. VC is an antioxidant found naturally in fruits, vegetables, and plants which can reduce the production of ROS. VC was shown to improve sudden sensorineural hearing loss [9] and protect hair cells against neomycin [10]. Based on these findings, we conceived the idea that VC could be a candidate for an otoprotective agent against neomycin-induced toxicity. The effects of VC on ototoxicity drug-induced hair cell damage and its potential function and mechanism need to be investigated.

The mechanisms of preventing aminoglycoside-induced ototoxicity include limiting cellular uptake of aminoglycoside and modulating cell death signaling pathways. In this study, we aimed to investigate neomycin-induced hair cell damage and to better understand the mechanism of VC effects on neomycin in hair cells. House Ear Institute-Organ of Corti 1 (HEI-OC1) cell line was used in this study. We investigated ROS production, apoptosis formation, and cell viability reduction in cells exposed to neomycin to determine whether VC inhibits neomycin-induced cell death. In addition, we explored the protective mechanism of VC against neomycin-induced cell death through inhibition of JNK activation and p38 phosphorylation. The results suggest that VC plays an important role in preventing neomycin-induced HEI-OC1 cell death and might be a potential therapeutic treatment of ototoxic drug-induced hearing loss.

## 2. Materials and Methods

### 2.1. Cell Line and Subsequent Experiments

HEI-OC1 cells were cultured in Dulbecco's modified Eagle's medium (DMEM) supplemented with 10% fetal bovine serum (FBS) at 33°C and 5% CO_2_ in a humidified atmosphere. Cell growth was approximately 80% confluent, and then, subsequent experiments were performed. The target neomycin concentration was determined and applied to HEI-OC1 cells to induce cell apoptosis; VC (0.5 mM) was applied with neomycin to the HEI-OC1 cells. Four groups were included in this study: HEI-OC1, HEI-OC1+ VC, HEI-OC1+ neo, and HEI-OC1+ neo+VC. HEI-OC1 only was a normal control group.

### 2.2. Concentration of Neomycin Assessment

To determine the concentration of neomycin-induced HEI-OC1 cell apoptosis, apoptosis markers including Bax, Bcl-2, Caspase-3, and Caspase-8 in neomycin-treated hair cells were detected by Western blot. After HEI-OC1 cell initial incubation, the experimental group was treated with different concentrations of neomycin at 0.5, 1.0, 2.0, and 5.0 mM for 24 h individually. The protein was detected by Western blot, and the measured expression levels were used to determine the appropriate concentration of neomycin according to neomycin-induced cell apoptosis. This neomycin concentration was used in subsequent experiments.

To determine the concentration of vitamin C, different concentrations of VC at 0.1, 0.2, 0.5, and 1 mM were applied to target concentration neomycin-treated HEI-OC1 cell for 24 h individually. The proteins of Bax and Bcl-2 were detected, and the measured expression levels were used to determine the appropriate concentration of VC.

### 2.3. Western Blot Analysis

We collected HEI-OC1 cells from the control and treated groups and then processed them for Western blot following standard protocols. In general, treated HEI-OC1 cells were washed and lysed with 180 *μ*l fresh buffer made up with Western and IP Lyse and PMSF on ice for 10 min, transferred to microcentrifuge tubes, and centrifuged at 12,000 × *g* for 10 min at 4°C. Supernatants were collected to quantify protein concentration using the BCA assay. Equal amounts of proteins (20 *μ*g) were loaded on and separated by 8%~15% SDS-PAGE electrophoresis and transferred to polyvinylidene fluoride membranes (PVDF). The membranes were blocked with 5% nonfat dried milk in TBS-T with 0.1% Tween-20 for 60 min at room temperature. The membranes were incubated overnight at 4°C with primary antibodies, including mouse anti-Bcl-2 (sc-7382, Santa Cruz, USA), rabbit anti-Bax (50599-2-ig, Proteintech), rabbit anti-cleaved-caspase3 (19677-1-AP, Proteintech), rabbit anti-caspase-8 (13423-1-AP, Proteintech), rabbit anti-AMPK*α* (5832S, CST, USA), rabbit anti-AMPK*α* (5832S, CST, USA), rabbit anit-JNK1 (ab179461, Abcam, USA), rabbit anti-P38 (8690S, CST, USA), rabbit anti-p-P38 (bs-5476R, Bioss, China), and mouse anti-GAPDH (60004-1-ig, Proteintech). In the next day, the membranes were washed with TBS-T and incubated with a secondary IgG antibody conjugated to horseradish peroxidase (HRP) (1 : 3,000) for 1 h. Immunoreactivity was detected with enhanced chemiluminescence (ECL) detection system. The intensity of protein bands was quantified using ImageJ software (Broken Symmetry Software, USA). Protein expression levels were normalized using GAPDH as the standard protein expression.

### 2.4. Cell Viability Measurement

Live cells were measured using the Cell Counting CCK-8 Kit (CCK-8, Meilunbio, China). HEI-OC1 cells were exposed to 2 mM neomycin and VC in 96-well plates. At 0, 1, 2, 3, and 4 days, experimental cells were incubated with 10 *μ*l of CCK-8 in each well for 30 min at 37°C, and a microplate reader (Bio-Rad) was used to measure the absorbance at 450 nm.

### 2.5. Cell Immunofluorescence Measurement

The cells were fixed by 4% paraformaldehyde for 30 min and then washed three times with PBS and incubated in blocking medium (goat serum, 1% Triton X-100) at room temperature for 30 min. The samples were incubated with the primary antibody Bax, Bcl2, caspase-3, and caspase-8 for 2 h at 4°C. After washing three times with PBST, the samples were incubated with the secondary antibody for 1 h at room temperature. The samples were washed and mounted on slides and were imaged with fluorescence microscope.

### 2.6. Flow Cytometry Analysis

HEI-OC1 cells were incubated with Annexin V Apoptosis Detection Kits (eBioscien, 88-8007-74) according to the manufacturer's instructions. The cells were collected, washed, and resuspended in 1× binding buffer at a concentration of 1 × 10^6^ cells/ml. A total volume of 5 *μ*l Annexin V-FITC and 5 *μ*l PI were added and mixed with 100 *μ*l cells and incubated for 15 min at room temperature. A total volume of 400 *μ*l 1× binding buffer was added to the tubes. The cells were analyzed by flow cytometry as soon as possible. Double-positive propidium iodide and Annexin-V staining was assigned as late-stage apoptotic cells, and double-negative staining was assigned as viable cells. The early apoptotic cells were stained with Annexin-V staining, and the necrotic cells were stained with PI only. Total apoptosis includes early and late-stage apoptosis.

### 2.7. TUNEL Staining Measurement

HEI-OC1 cell apoptosis was determined by TUNEL staining. The TUNEL assay was conducted using the Click-iT® Plus TUNEL Assay (Life Technologies, USA) according to the manufacturer's protocol. Briefly, HEI-OC1 cells that were given the indicated treatment were fixed with 4% PFA in PBS for 30 min at room temperature and then washed with PBS. The cells were permeabilized with 0.1% Triton X-100 in PBS for 10 min and then stained with TUNEL working solution for 1 h at 37°C in dark. Cell nuclei were stained with DAPI. The specimens were visualized using a confocal microscope.

### 2.8. ROS Detection

DCFH-DA (D6883, Sigma Technologies) staining was used to assess cellular ROS levels according to the manufacturer's instructions. Briefly, the cells were seeded in 6-well plates at a density of 2.5 × 10^5^ cells/well and treated with the designated conditions. After washing with prewarmed serum-free DMEM, the cells were incubated with 10 *μ*M DCFH-DA in serum-free medium for 30 minutes, followed by washing twice with PBS. The intensity of the fluorescent signal was determined with fluorescence microscopy.

### 2.9. Statistical Analysis

Data were statistically analyzed with one-way ANOVA and two-way ANOVA when comparing more than two groups followed by the Tukey multiple comparison post hoc test using GraphPad Prism. The unpaired *t*-test was used to compare between two groups. The data are shown as the mean ± SD. *p* < 0.05 was considered a statistically significant difference.

## 3. Results

### 3.1. Neomycin-Induced HEI-OC1 Cell Apoptotic Protein Expression Is Concentration-Dependent

**Figure 1:**
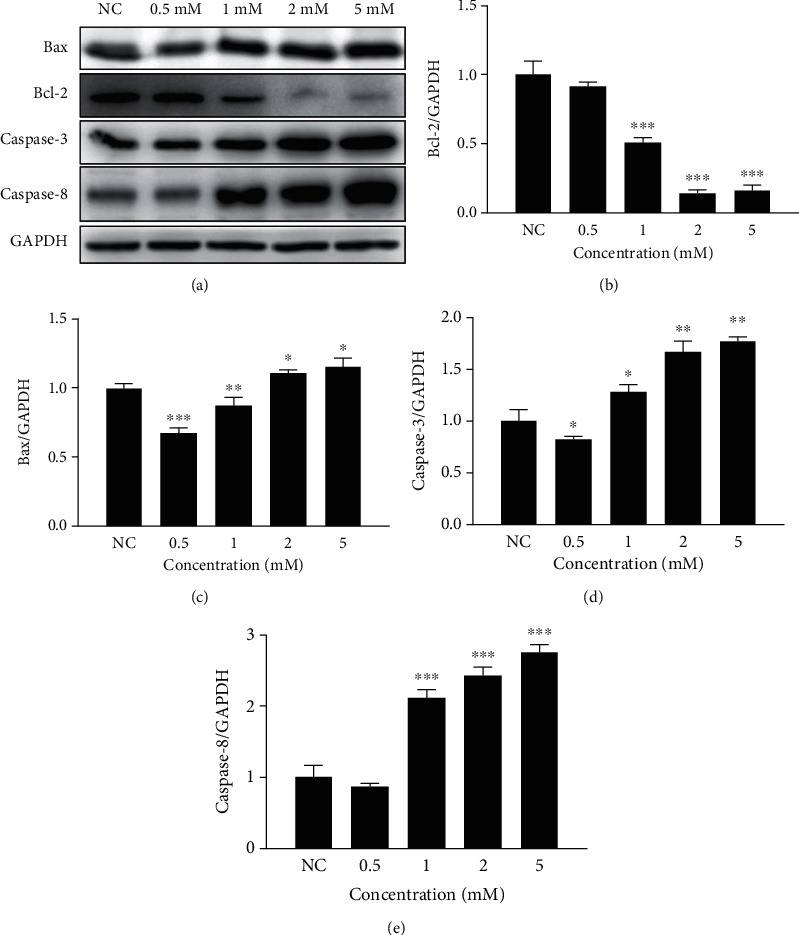
Neomycin-induced apoptotic protein expression at different concentrations. (a) Four apoptotic-related protein (Bax, Bcl2, caspase-3, and caspase-8) expressions were detected by Western blot in HEI-OC1 cells treated with 0.5, 1.0, 2.0, and 5.0 mM neomycin. The expression levels of Bcl-2 (b), Bax (c), caspase-3 (d), and caspase-8 (e) were normalized to GAPDH at different concentrations of neomycin. One-way ANOVA, ^∗^*p* < 0.05, ^∗∗^*p* < 0.01, and ^∗∗∗^*p* < 0.001, versus the normal control. All tests were repeated three times independently.

To determine the appropriate concentration of neomycin-induced HEI-OC1 apoptosis, four different concentrations of neomycin were selected: 0.5, 1.0, 2.0, and 5.0 mM. Apoptosis-related proteins were detected by Western blot (Figure 1(a)). The protein expression level of neomycin-induced apoptosis was evaluated to determine the experimental target concentration. Apoptotic protein expression levels of Bax, Caspase-3, and Caspase-8 were increased, and antiapoptotic protein Bcl-2 expression level was decreased relative to the neomycin concentration increase (Figures 1(b)–1(e)). The dose-dependent expression level shows the strongest apoptotic reaction change among all four proteins treated with 2.0 mM neomycin. Therefore, 2.0 mM neomycin was selected as the target concentration, and this concentration was used for the following experiments.

**Figure 2:**
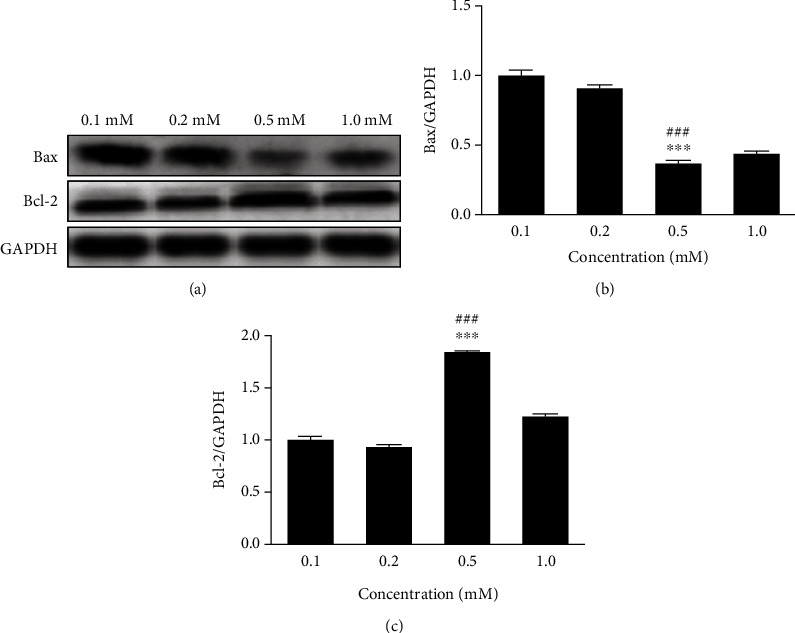
Neomycin-induced apoptotic protein expression with different concentrations of VC treatment. (a) Protein expression of Bax and Bcl2 was detected by Western blot in neomycin-treated HEI-OC1 cells with 0.1, 0.2, 0.5, and 1.0 mM VC individually. The expression levels were normalized to GAPDH (b and c). One-way ANOVA, ^∗∗∗^*p* < 0.001 versus 0.1 mM group and ^###^*p* < 0.001 versus 1.0 mM group. All tests were repeated three times independently.

To determine the appropriate concentration of VC, four different concentrations of VC at 0.1, 0.2, 0.5, and 1.0 mM were applied to 2.0 mM neomycin-treated HEI-OC1 cell. The protein expression levels of Bax were decreased, and Bcl-2 expression level was increased, and the strongest apoptotic reaction change was detected at the concentration of 0.5 mM VC treatment (Figure 2). Therefore, 0.5 mM VC was selected as the target concentration.

### 3.2. VC Treatment Increased the Viability of HEI-OC1 Cells Exposed to Neomycin

**Figure 3:**
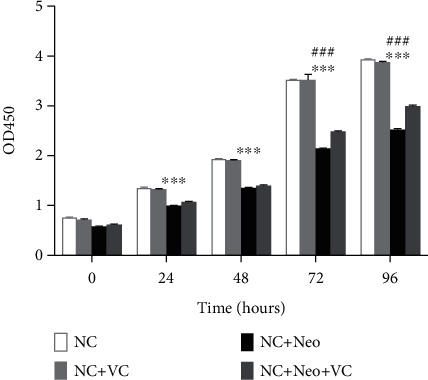
Cell viability treated with neomycin and VC. Cell alive rates were examined using the CCK-8 cell counting kit. Four group cell viabilities were measured at 24, 48, 72, and 96 hours. Detection absorbance at 450 nm was determined at the time points. Two-way ANOVA, ^∗∗∗^*p* < 0.001 versus NC group and ^###^*p* < 0.001 versus NC+Neo+VC group. All tests were repeated three times independently.

Cell viability at different time points was measured with a CCK-8 assay. In HEI-OC1 cells treated with VC, the cell viability was similar to the control group and did not affect HEI-OC1 cell viability. When HEI-OC1 cells were treated with 2 mM neomycin, cell viability decreased with time duration, indicating that neomycin affected cell viability. When VC was applied with neomycin to treat the HEI-OC1 cells, cell viability gradually increased with time duration compared to the neomycin-treated cells, and the viability rate was increased after 72 hours (Figure 3). The results showed that VC ameliorated neomycin-decreased HEI-OC1 cell viability, thus indicating that VC had protective effects on HEI-OC1 cell viability.

### 3.3. VC Treatment Attenuated Neomycin-Induced HEI-OC1 Cell Death

**Figure 4:**
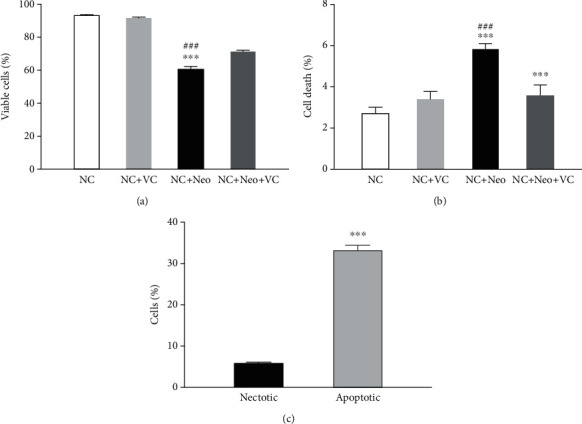
Percentage of viable and damaged cells treated with VC and neomycin. Viable and dead cells were measured with flow cytometry. (a) Percentage of viable cells in various treatment groups. (b) Percentage of death cells with neomycin and VC treatment. One-way ANOVA, ^∗∗∗^*p* < 0.001 versus NC group and ^###^*p* < 0.001 versus NC+Neo+VC group. (c) Percentage of necrotic and apoptotic cells exposed to neomycin, two-tailed, unpaired *t*-test, ^∗∗∗^*p* < 0.001. All tests were repeated four times independently.

Neomycin exposure decreased HEI-OC1 cell viability. Treated HEI-OC1 cells were detected by flow cytometry, and the percentage of death and survival cells was calculated. The percentage of cell death and survival was not affected by VC treatment. The percentage of survival HEI-OC1 cells was decreased after neomycin exposure and increased with simultaneous treatment with neomycin and VC (Figure 4(a)). The results showed that the HEI-OC1 cell death was increased with neomycin treatment compared to normal control and VC treatment groups. The HEI-OC1 cell death was reduced with VC and neomycin cotreatment compared to neomycin treatment only (Figure 4(b)). Neomycin-induced apoptosis and necrosis, total apoptotic cells, and necrotic cells increased with neomycin exposure, and the percentage of total apoptotic cells was much greater than that of necrotic cells (Figure 4(c)), suggesting that apoptosis is mainly neomycin-injured cells.

### 3.4. VC Ameliorated Neomycin-Induced ROS Generation in HEI-OC1 Cells

**Figure 5:**
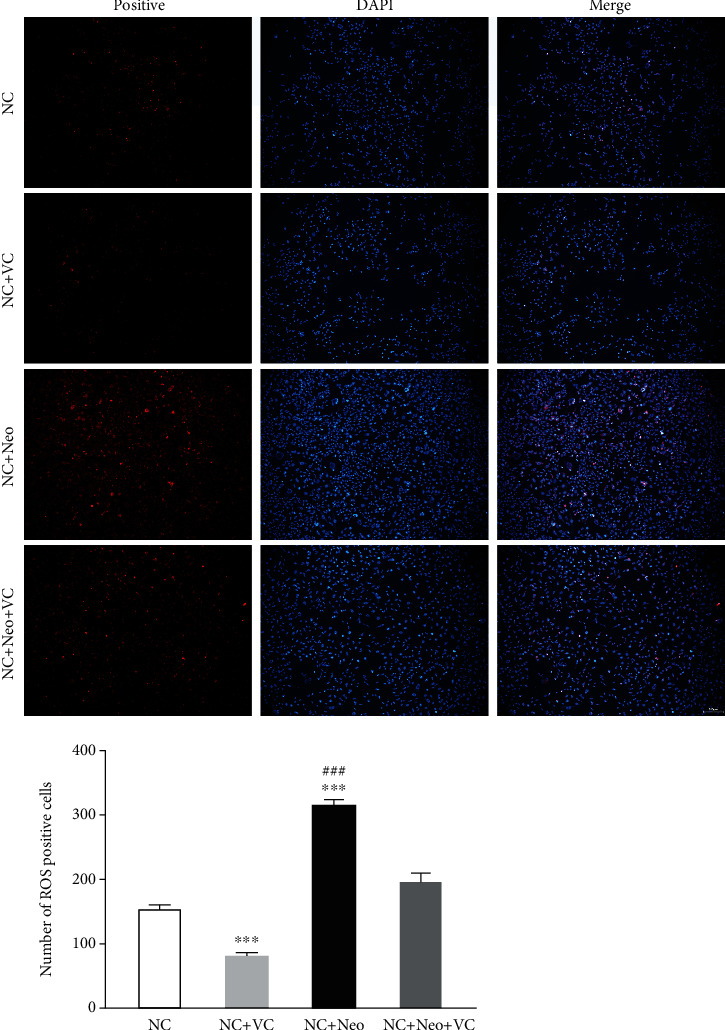
Expression of ROS on neomycin and VC-treated HEI-OC1 cells. ROS of HEI-OC1 cells was detected by DCFH-DA staining. The ROS-positive staining is red, scale bar = 100 *μ*m. The expression of staining on normal control cells, VC-treated cells, neomycin-treated cells, VC, and neomycin-cotreated cells. The number of ROS-positive staining cells with neomycin and VC treatment. One-way ANOVA, ^∗∗∗^*p* < 0.001 versus NC group and ^###^*p* < 0.001 versus NC+Neo+VC group. All tests were repeated three times independently.

We investigated the effect of VC on neomycin-induced ROS in HEI-OC1 cells. HEI-OC1 cells treated with VC exhibited less ROS-positive staining compared to the control cells, indicating that VC decreased ROS generation in cells. Intracellular ROS in cells treated with neomycin were increased compared to the normal control. Coadministration VC with neomycin inhibited neomycin-induced ROS formation (Figure 5), which indicates that VC protected against neomycin-induced ROS production.

### 3.5. VC Treatment Reduced Neomycin-Induced HEI-OC1 Cell Apoptosis

**Figure 6:**
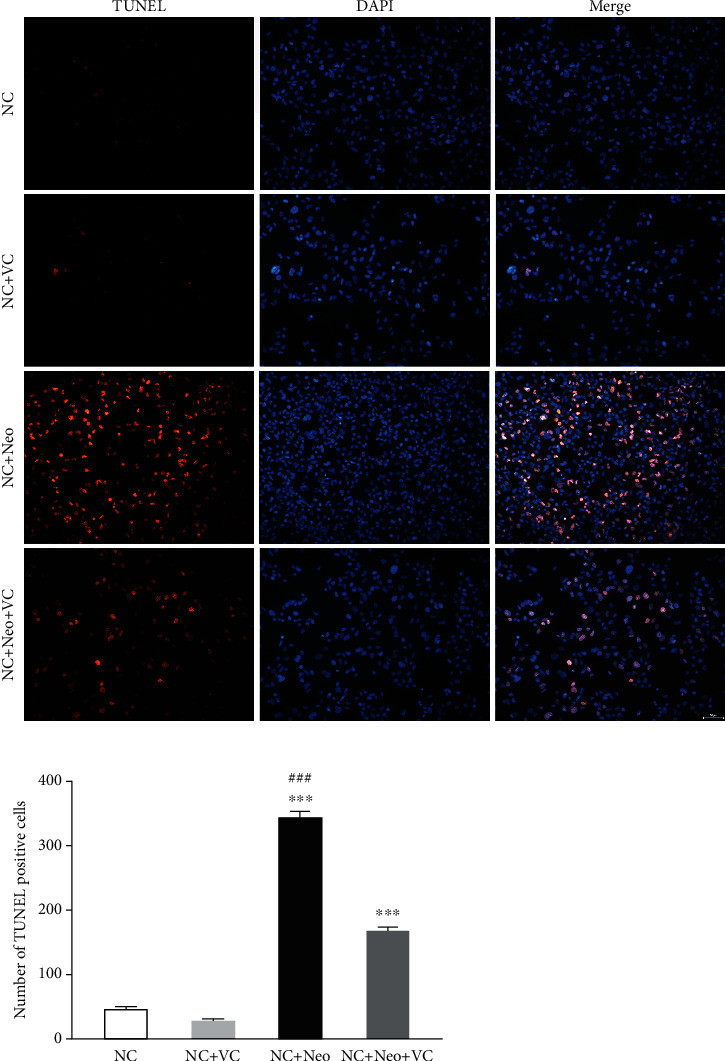
Apoptosis expression in VC and neomycin treated HEI-OC1 cells. The apoptotic cell was detected using TUNEL staining. The TUNEL staining is red and expressed at different treatment groups, scale bar = 50 *μ*m. The number of positive TUNEL staining cells with different treatment. One-way ANOVA, ^∗∗∗^*p* < 0.001 versus NC group and ^###^*p* < 0.001 versus NC+Neo+VC group. All tests were repeated three times independently.

The TUNEL assay and DAPI staining were performed to determine neomycin-induced HEI-OC1 cell death by apoptosis, in addition to whether this could be prevented by VC treatment. As shown in Figure 6, neomycin treatment increased TUNEL-positive cells, and VC and neomycin cotreatment decreased TUNEL-positive cells. The cells of control and VC only without neomycin showed fewer positive cells. The results confirmed that neomycin promoted apoptotic cell death, and VC inhibited neomycin-induced HEI-OC1 cell apoptosis.

**Figure 7:**
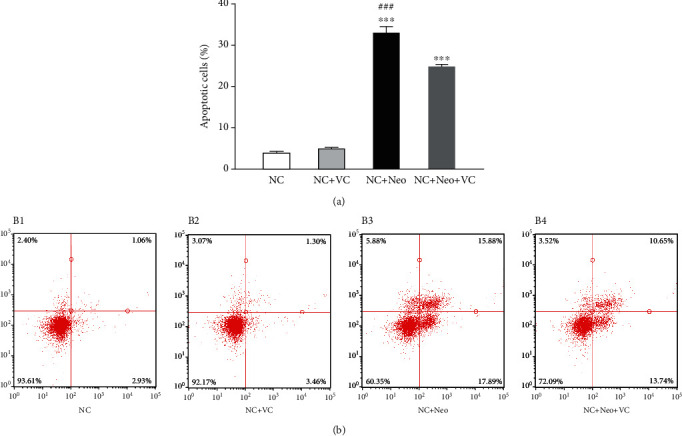
VC effects on neomycin-induced HEI-OC1 cell apoptosis. Flow cytometry of Annexin V-FITC and PI double-stained HEI-OC1 cells were assigned as apoptosis. Total apoptosis includes early and late-stage apoptosis. Quantification of the percentage of apoptosis is shown (a). One-way ANOVA, ^∗∗∗^*p* < 0.001 versus NC group and ^###^*p* < 0.001 versus NC+Neo+VC group. Histograms of four groups were exhibited (b1–b4). Neomycin increased early (LR) and late apoptosis (UR), and neomycin-induced apoptosis was inhibited by cotreatment with VC. All tests were repeated four times independently.

Verification and quantification of the number of apoptotic cells induced by neomycin-treated HEI-OC1 cells were accomplished using flow cytometry. The percentage of apoptotic cells treated with neomycin or cotreatment with VC was analyzed by using Annexin V-FITC and PI double staining. The number of apoptotic cells counted as late apoptotic cells was shown in the upper right quadrant (UR), and early apoptotic cells were plotted in the lower right quadrant (LR) of the histograms (Figures 7(b1)–7(b4)). The number of total apoptotic cells (UR+LR) with neomycin plus VC (mean 24.7%) was reduced compared to neomycin-only (mean 33.2%, *p* < 0.001) (Figure 7(a)). The results showed that neomycin promoted apoptotic cell death (*p* < 0.001), and this was inhibited by simultaneous VC coadministration.

### 3.6. VC Suppressed Neomycin-Induced HEI-OC1 Cell Apoptotic Protein Expression

**Figure 8:**
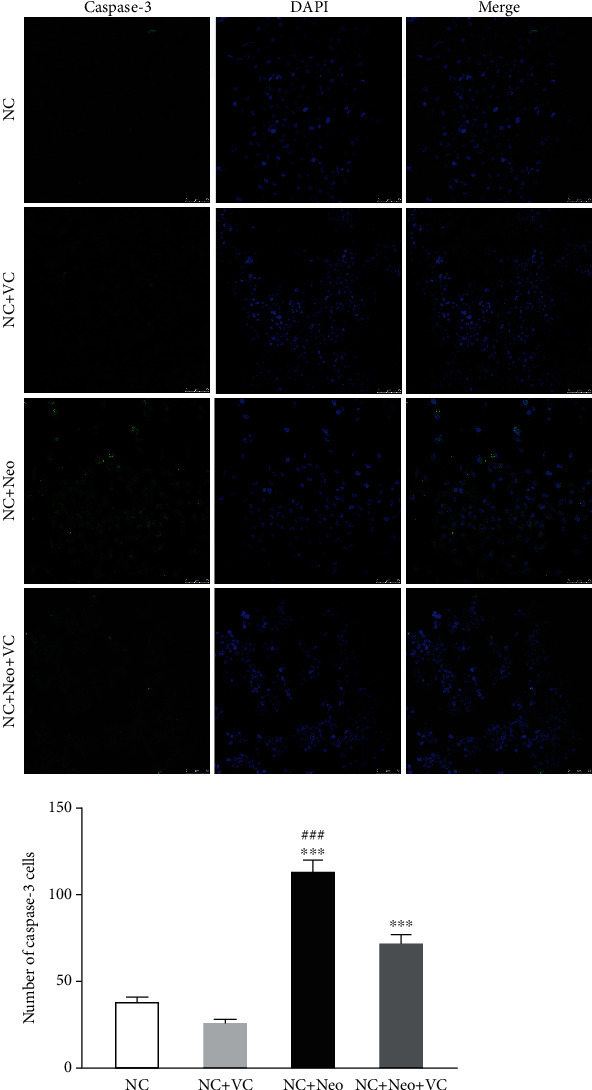
The expression of caspase-3 on VC and neomycin-treated HEI-OC1 cells. Caspase-3 was detected by immunostaining (green), scale bar = 10 *μ*m. The caspase-3-positive staining cells were observed after neomycin exposure compared to the control and VC groups. The number of positive staining cells shows a significant difference with different treatment. One-way ANOVA, ^∗∗∗^*p* < 0.001 versus NC group and ^###^*p* < 0.001 versus NC+Neo+VC group. All tests were repeated three times independently.

**Figure 9:**
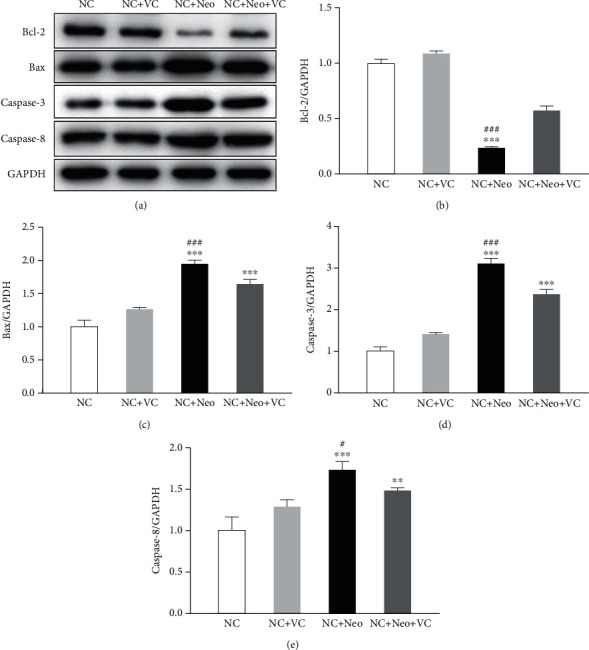
Apoptotic-related protein expression in treated HEI-OC1 cells. The expression of the apoptotic protein was detected by Western blot (a). Bcl-2, Bax, caspase-3, and caspase-8 proteins were detected. The protein expression level was measured with each band gray value, normalized with GAPDH (b–e). One-way ANOVA, ^∗∗^*p* < 0.01 and ^∗∗∗^*p* < 0.001 versus NC group and ^#^*p* < 0.05 and ^###^*p* < 0.001 versus NC+Neo+VC group. All tests were repeated three times independently.

To assess the VC effect on neomycin-induced HEI-OC1 cells, apoptotic-related proteins were detected after cells were administrated neomycin and VC. As apoptosis executioner molecules, caspase-3 was detected by immunostaining to assess cell apoptosis associated with neomycin-induced ototoxicity [11].  Figure 8 shows that caspase-3 levels increased after neomycin exposure and decreased with VC and neomycin cotreatment. The protein expressions of apoptosis of Bax, Bcl-2, caspase-3, and caspase-8 were detected by Western blot.  Figure 9 shows neomycin treatment decreased the expression level of Bcl-2, and VC inhibited the decrease in the expression level. Neomycin treatment resulted in increased protein expression of Bax, cleaved caspase-3, and caspase-8, and VC treatment blocked the increased expression of Bax and cleaved caspase-3. The protein expression illustrated that the effect of VC on the neomycin-induced apoptotic response is protective against neomycin-induced HEI-OC1 cell apoptosis related to Bax, Bcl-2, and caspase-3.

### 3.7. VC Mediated Activation of JNK1 and Phosphorylation of p38 Effect on Neomycin-Induced HEI-OC1 Cell Apoptosis

**Figure 10:**
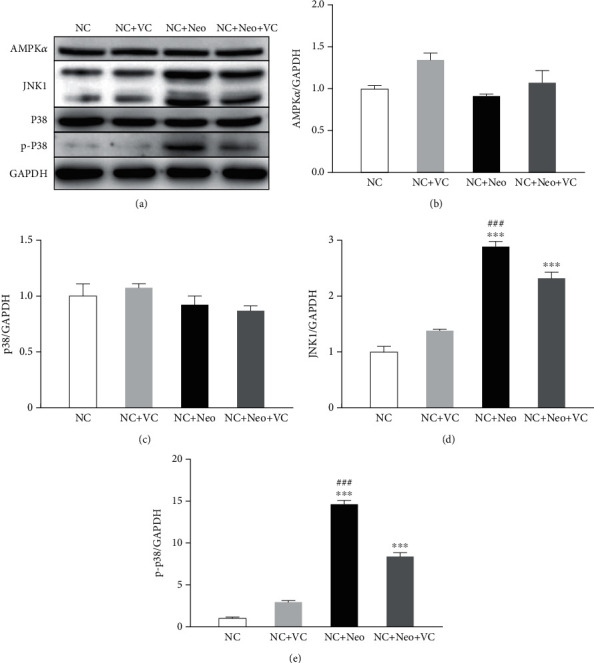
VC-related neomycin-induced HEI-OC1 cell apoptosis pathway. Pathway proteins related to apoptosis were detected. The protein expression was detected by Western blot. The expressions of AMPK*α*, JNK1, p38, and p-P38 were detected (a). The protein expression level was measured with each band gray value, normalized to GAPDH (b–e). One-way ANOVA, ^∗∗∗^*p* < 0.001 versus NC group and ^###^*p* < 0.001 versus NC+Neo+VC group. All tests were repeated three times independently.

To better understand the effects of VC on neomycin-induced HEI-OC1 cell apoptosis, we detected signaling pathway proteins related to apoptosis.  Figure 10 shows that the expression of AMPKa and p38 was not different between the treated and untreated groups. The protein expression level of JNK1 was increased after neomycin exposure and decreased after VC was applied simultaneously. The same pattern was also seen in Figure 10, which demonstrates that phosphorylated p38 was activated after neomycin treatment, and VC treatment inhibited neomycin-induced p38 phosphorylation. These results illustrated that VC inhibited JNK1 activation and p38 phosphorylation to protect against neomycin-induced cell apoptosis.

## 4. Discussion

To better investigate the mechanism of hair cell damage under neomycin exposure, an appropriate cell line is important for drug-induced cytotoxicity. House Ear Institute-Organ of Corti 1 (HEI-OC1) cells are a mouse auditory cell line derived from the auditory organ of a transgenic mouse [12]. This cell line expresses markers for auditory sensory cells and was used to investigate the aminoglycoside-induced ototoxic mechanism [13, 14]. In this study, we used HEI-OC1 cells to investigate the effect of VC on neomycin-inducted cell ototoxicity. We screened different neomycin concentrations applied to induce specific apoptosis related proteins in the HEI-OC1 cell and showed that the neomycin-induced apoptotic reaction was dose-dependent, with 2 mM neomycin causing the strongest reaction. For the experiment, a 2 mM target concentration was used to investigate neomycin-induced cell damage and VC protective effect on cell damage.

ROS generation has been associated with ototoxic drug-induced hair cell damage [15], and neomycin induces ROS production in hair cells. ROS is a major factor of aminoglycoside-induced hair cell apoptosis [16, 17]. In this study, the results support that neomycin induced ROS elevation and decreased cell viability. The results indicated that high ROS concentrations contribute to HEI-OC1 cell death. ROS-triggered cell death mechanisms were related to caspase-dependent and independent apoptosis, as well as necrosis. In contrast to other aminoglycosides that do not promote HEI-OC1 cell death [13], our results showed neomycin-induced cell death, and apoptosis is mainly responsible for neomycin-induced cell death. Apoptosis is regulated by multiple genes and enzymes, including the Bcl-2 and caspase families [18]. Caspases are a family of cysteine proteases that participate in the apoptosis process by triggering apoptosis signaling. Aminoglycosides can activate caspase in the hair cell line [14]. Our Western blot analysis showed that the apoptosis-related genes Bcl-2 and Bax altered by oxidative stress influence mitochondria to initiate cell apoptosis. Apoptosis also occurs through initiator caspase-8 and executioner caspase-3 to cause hair cell death. The present study indicated that neomycin-induced HEI-OC1 cell cytotoxicity occurs via ROS generation, with caspase-8 triggered and caspase-3 activated protein cleavage, leading to apoptosis as the primary type of cell death. A ROS-triggered cell death mechanism with consequent apoptosis promoting, Bcl-2 and caspase family proteins were both involved in this process. Together, these results suggest that ROS contribute to cell death as they are generated during the apoptotic process [4, 19].

Antioxidants also protect against ototoxicity by reducing ROS levels. Antioxidants show otoprotective effects in animal models of aminoglycoside ototoxicity and can ameliorate gentamicin-induced ototoxicity in the clinic [20, 21]. VC is a water-soluble and basic dietary requirement for humans. It is well known for its role in preventing scurvy [22]. Unlike other mammals and animals, humans are not able to synthesize VC in the body because of the mutation in the gene encoding L-gluconolactone oxidase [23]; hence, VC must be obtained from their daily diet or dietary supplement. VC is a protective agent against ROS induced by neomycin. Previous reports showed that VC as an antioxidant reduces noise and drug-induced ototoxicity and prevents spiral ganglion neuron degeneration [9, 24, 25]. In the present study, the VC protective effect on neomycin-induced HEI-OC1 cell damage was investigated. The ROS results demonstrated that VC is a highly effective antioxidant that attenuated neomycin-induced ROS production in HEI-OC1 cells, and VC had ROS reduced effect on normal cells at the same concentration. In association with antioxidant activity, VC can affect cell viability. Present studies showed that neomycin and VC treatment with distinctive doses have time-dependent and time-sensitive effects on cell viability. The cell viability study demonstrated that VC decreased neomycin-induced cell death, and VC had no effect on normal cell viability. In addition, we investigated the contribution of VC to neomycin-induced hair cell apoptosis. In contrast to the synergism with cisplatin in promoting carcinoma apoptosis [26], our study demonstrates that VC had a reverse effect on neomycin-induced apoptosis, with VC suppressing the apoptosis signaling cascades. We examined the expression of apoptosis-related protein in cells treated with neomycin and VC, showing that VC prompted antiapoptotic Bcl2 expression in HEI-OC1 cells exposed to neomycin and inhibited proapoptotic Bax, cleavage of caspase-3, and caspase-8 expression when exposed to neomycin. The results suggested that VC influenced neomycin-induced HEI-OC1 cell death via extrinsic and intrinsic apoptosis pathways, indicated that VC effectively inhibited neomycin-induced apoptosis, and revealed that VC protects against neomycin-induced ROS formation.

Furthermore, we investigated the factors involved in VC opposed to neomycin-induced HEI-OC1 cell death. Previous studies have shown that JNK was activated by ROS which formed during oxidative stress [8, 27], associated with neomycin-induced apoptosis via p38, MAPK, and JNK [6] activation in hair cells. JNK activation can be inhibited by antioxidants [28]. p38 inhibition reduced radiation-induced activation of JNK, p38, and cleavage of caspase-3 in HEI-OC1 cells [29]. Different from previous studies, the present study shows that VC cotreatment suppresses proapoptotic protein expression levels mediated by p38 phosphorylation and JNK activation and that p38 and AMPK were not activated. These results suggest that the downregulation of JNK and p38 signaling pathways plays an important role in VC suppression of neomycin-induced apoptosis in HEI-OC1 cells. These results provide an understanding of the mechanism of VC's protective effects on neomycin-induced HEI-OC1 cell death.

## 5. Summary

Neomycin causes HEI-OC1 cell death by increased ROS and induced cell apoptosis. VC ameliorated HEI-OC1 cell death from neomycin-induced ROS production, inhibited apoptosis activation, and attenuated cell damage. VC inhibited apoptosis by suppressing JNK activation and p38 phosphorylation. These results indicate that VC may be used as a protective factor to decrease the ototoxicity of neomycin. Further studies are ongoing to define the effect of VC on systematic administration in vivo.

## Data Availability

The data used to support the findings of this study are available from the corresponding author upon request.
